# Global human obesity and global social index: Relationship and clustering

**DOI:** 10.3389/fnut.2023.1150403

**Published:** 2023-03-09

**Authors:** Mubbasher Munir, Zahrahtul Amani Zakaria, Haseeb Nisar, Zahoor Ahmed, Sameh A. Korma, Tuba Esatbeyoglu

**Affiliations:** ^1^Faculty of Informatics and Computing, University of Sultan Zainal Abidin, Terengganu, Malaysia; ^2^Department of Economics and Statistics, University of Management and Technology, Lahore, Pakistan; ^3^Department of Life Sciences, University of Management and Technology, Lahore, Pakistan; ^4^Department of Human Nutrition and Dietetics, School of Food and Agricultural Sciences, University of Management and Technology, Lahore, Pakistan; ^5^Department of Food Science, Faculty of Agriculture, Zagazig University, Zagazig, Egypt; ^6^School of Food Science and Engineering, South China University of Technology, Guangzhou, Guangdong, China; ^7^Department of Food Development and Food Quality, Institute of Food Science and Human Nutrition, Gottfried Wilhelm Leibniz University Hannover, Hannover, Germany

**Keywords:** globesity, global social index, human development index, clustering, global happiness, globalization, sustainability, political globalization

## Abstract

**Introduction:**

Obesity, a complex, multifactorial disease, is considered a global disease burden widely affecting the quality of life across different populations. Factors involved in obesity involve genetics, behavior and socioeconomic and environmental origins, each contributing to the risk of debilitating morbidity and mortality. However, the trends across the world vary due to various globalization parameters.

**Methods:**

This article tends to identify the global social indicators, compiled into a global index, and develop a correlation between the global social index created by using the human development index, social and political globalization, the global happiness index, and the quality of infrastructure, institutions, and individuals using the internet factors and its effect on global obesity.

**Results and Discussion:**

Our results identified a positive correlation between medium human development levels with obesity compared to low and very high human development levels. Economic stability due to rapid industrialization has increased the buying capacity and changed the global food system, which seems to be the major driver of the rise of global obesity.

**Conclusion:**

The results decipher that global social indicators and overall social index have positively affected global obesity, which will help policymakers and governmental organizations monitor the obesity patterns across their regions by a significant contribution from globally influenced social factors.

## 1. Introduction

Obesity is a medical condition characterized by having an excessive amount of body fat that can negatively impact a person’s health. It is typically defined as having a body mass index (BMI) of 30 or higher, but other factors such as waist circumference and body composition may also be considered. Obesity can increase the risk of various health problems, including cardiovascular disease, type 2 diabetes, and certain types of cancer. It can be caused by a variety of factors, including genetics, lifestyle habits, and environmental influences (1, 2). These diseases lead to premature deaths, making obesity one of the leading factors of worldwide mortality (3). Global obesity prevalence is anticipated to reach 18% in men and exceed 21% in women by 2025, with severe obesity exceeding 6% in men and 9% in women (4).

It is pertinent to mention that global obesity patterns vary ethnically. Its trend changes from one part of the world to another, e.g., Black African women, Black Caribbean abdominal obesity (fat localized mainly in the abdominal region) than the general population (5). It is difficult to understand why these variations in obesity patterns prevail across different races and ethnicities; nevertheless, genetic variations and cultural body image differences seem to be important factors that drive these changes (6, 7). Generally, economic wealth and obesity patterns have a direct relation, yet variations in this correlation pattern remain to be answered (8). This can be seen from the fact that economically prosperous countries of East Asia, e.g., (South Korea, Singapore, and Japan) have the lowest obesity prevalence around the world.

In contrast, the Middle East and nations in Latin America with much lower levels of economic development have significant obesity rates. Cultural influences may affect behavioral decisions regarding nutrition and physical exercise in response to the increasingly obesogenic environment brought on by economic affluence. Individual choices, however, are not autonomous, including selecting to eat specific foods in certain amounts, to follow a particular lifestyle or to exercise and how much. The cultural influence and its characteristics at the societal level affect personal behavior and may contribute to the variance in obesity rates between countries (9). Individuals dietary and behavioral decisions might lessen the impact of the environment on their body weight. The two dimensions of societal type, i.e., individualism and collectivism, also have different impacts on obesity patterns. The individualism-type societies, like in Western Europe, allows the freedom of its members to live their life according to their wishes and are free to manage their consumption habits without significant interference from any other member (10). Contrarily, societies that work on collectivism, like low-income African countries, and Middle Eastern and South Asian countries, impose social restrictions on many features of an individual life, including food consumption (11). It has been studied that people from low- and middle-income countries have a close correlation between food insecurity and climate change vs. obesity, while people from developed countries have an increasing trend in obesity due to increased greenhouse gases, agricultural production and industrialization (12, 13). The World Health Organization also advises that measures be taken to prevent abdominal obesity, particularly for members of ethnic groups who are greater propensity to develop metabolic syndrome, a collection of heart disease and other health issues like diabetes, such as those of South Asian descent.

Various factors are responsible for the variations in this global obesity pattern, most notably health and food quality, economic development, environment and genetic structure (14). However, economic wealth, prosperity and open trade practices are considered the predominant factor that provides increasingly abundant processed food and aggressively marketed food affordable to the ever-increasing global population (15). Further, it is also reported that the impact of migration on obesity in different ethnic groups is differentiated, with migrant groups having markedly higher obesity levels than the natives of that particular area (16). It was suggested that external factors like diet, lifestyle and urbanization have an important role in a probable association between migration and increased obesity. It is often assumed, rather than objectively measured, the role of cultural practices and beliefs in the hypothesis of why obesity patterns are varied across different ethnicities. This may be presented by the fact that people from South Asian backgrounds have increased cholesterol consumption in their diets (17) and less physical activity (18). Perceptions of a population’s potential advantages of modern lifestyles, such as increasing automobile use and lower calorie expenditures, as well as foreign diets (e.g., which may increase calorie consumption through intake of fast food rich in fats and sugars) perceptions of a population’s potential advantages of modern lifestyles, such as increasing automobile use and lower calorie expenditures, as well as foreign diets (e.g., which may increase calorie consumption through intake of fast food rich in fats and sugars), may have increased as a result of social and cultural globalization, which includes cross-border cultural shifts and media impact (19).

Through this analysis, we aim to determine whether the social dimension, an important part of overall globalization plays a greater or lesser part in raising the risk of obesity. Globalization has contributed to the rise in obesity rates around the world through factors such as increased availability of processed and high-calorie foods, changes in dietary habits, and reduced physical activity due to changes in work and transportation patterns. However, other factors such as genetics, culture, and individual behavior also play a role in obesity (20, 21). Recently the effects of globalization were measured on three different dimensions, i.e., (1) economic, (2) political, and (3) social, using the KOF globalization index measures in three main dimensions of global perspectives (22), which measures each of these dimensions in a precise way. Geographically, these components might contribute to obesity differently by stimulating increased calorie consumption and reduced energy consumption. The relationship between globalization and technological drift is often coherent and needs to be studied in parallel with each other. This can be elaborated with the notion that with technological development, globalization is also changed; hence both have similar factors linked with obesity collectively termed as “nutritional transition” or shifting from plant-based diets towards red meat and processed foods causing high-calorie intake resulting in weight gain and chronic illness (23, 24).

Political globalization indirectly plays a role in an increased obesity pattern. This can be stated by the creation of regional trade blocks, Asian pacific economic cooperation (APEC), or involvement of various multinational treaties that acts as a precursor for increased economic activities, including opening various markets and food chains that consequently result in associated overweight. This idea is supported by numerous data showing that political and economic globalization, particularly global trade expansion, has contributed to global obesity due to the ease of movement of obesogenic products like high-caloric beverages and packed foods to developing countries (25, 26). This can be justified by results from an independent study of 300 medical students from Iran that showed a significant association between fast-food consumption with abdominal obesity (27). Even though it would be difficult to understand the precise effect of such political effects on global obesity, it can be considered that political globalization acts independently or as a promoter *via* economic forces (21). A recent study from Munir et al. has shown that a low level of political globalization tends to increase global obesity while contrasting results were observed in areas with a high level of political globalization (28).

Social and cultural globalization involves cross-border cultural exchanges, and media transparency have changed the perception of conventional people with the rapid adaptation of western lifestyles (like increased use of cars, reduced calorie expenditures and increased uptake of junk foods). The effect of social globalization on overweight can be linked to rapid urbanization related to either increased calorie uptake (29, 30) or ample supply and consumption of energy-rich diets (31).

The effect of global happiness is highly variable in determining its relationship with obesity, and this is because various determinants influence differently with happiness. The non-linear relationship between obesity and happiness can be observed where happiness first increases with an increase in body mass index, then stabilizes and decreases after reaching the threshold body mass index. Obesity directly influences factors like physical appearance, health status and income which play a significant role in happiness (32). People with beautiful appearances are more likely to receive praise and compliments, which may bring higher levels of subjective happiness (33).

Similarly, health is associated with subjective happiness, as healthy individuals are more likely to withstand challenges and be involved in active lifestyles, which may benefit happiness (34). Contrarily, poor health leads to weak emotions, psychosocial pain and low self-esteem, which harm happiness (35). The relation of income with happiness is directly related since it has been seen in various countries that income was significantly positively associated with happiness (36). In addition, obese persons also have less chance of employment and are at higher risk of losing their job (37).

Transportation options and neighborhood walkability can be influenced by infrastructure, which is connected to a person’s weight status. A low prevalence of overweight and obesity is associated with high neighborhood walkability (38). Similarly, low physical activity level is increased by inadequate community designs and infrastructure that includes few safe walkways, bicycle paths and playgrounds (39). Exploring the factor structure of the Perceived Stress Scale in a sample of Chinese nurses: a principal axis factoring analysis used in the Journal of Advanced Nursing, which used PAF to explore the factor structure of the Perceived Stress Scale in a sample of Chinese nurses.

This study aims to determine whether socialization, an important dimension of overall globalization, plays a greater or lesser part in raising the risk of being overweight and human obesity. This objective was achieved *via* three methodologies, i.e., (1) development of a global social index through factor axis factoring, (2) develop relationship and clustering, and (3) checking the impact of global social indicators (individual and combined both) on global obesity.

## 2. Materials and methods

### 2.1. Data collection

Collection of all possible global available databases like Globalization index (KOF), world development indicators (WDI) by the World Bank, World Health Organization (WHO), and Food and Agriculture Organization (FAO),^1^ Our world in data (OWD) and several global data depositaries, from 183 countries and the period 2008–2019 are targeted to develop Global social index. Unique databases using complete indicators are merged using Principal axis factoring through R language. Missing observation analyses have been performed to enhance more efficiency of statistical results.

### 2.2. Formation of social index

**Figure 1:**
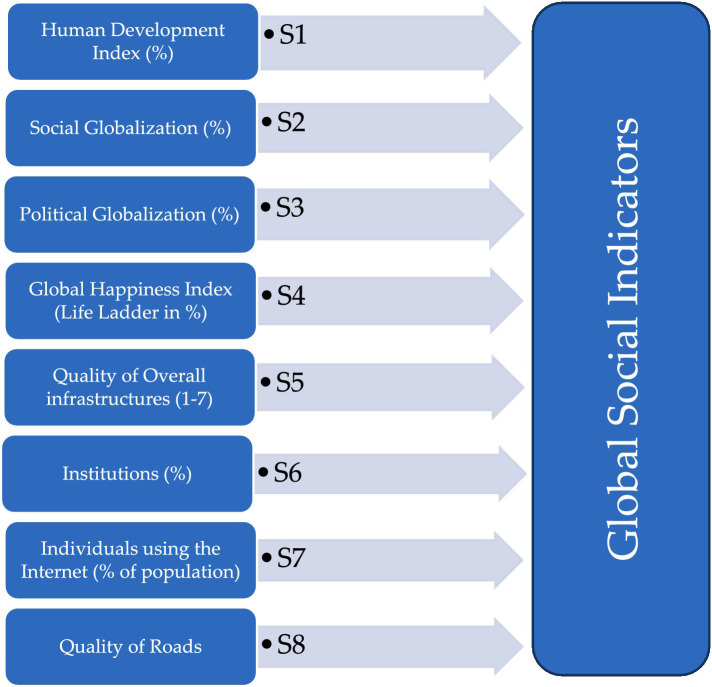
Identified eight social index indicators.

A social construct is one of the essential factors in the universe that contain several variables that directly or indirectly affect human obesity. For this purpose, nine global variables were used for the formation of a social index in the below flowchart, but “Poverty headcount ratio at $1.90 a day (2011 PPP) (% of the population)” was removed due to more missing values. Finally, eight global databases, including the Human Development Index (%), Social Globalization (%), Political Globalization (%), Global Happiness Index (Life Ladder in %), Quality of Overall infrastructures (1–7), Institutions (%), Individuals using the Internet (% of population) and Quality of Roads (1–7) have been used to construct seven social Indicators (S1–S8: Figure 1). These were used relevant and most recent databases in the context of social development and socialization in global prospective. In the previous studies, these indicators have been analyzed individually with varied results. In this study, all the indicators have been combined to see the effect on human obesity.

Principal axis factoring (PFA) is used to create index of multiple variables from global valid datasets. PAF aims to find a new set of uncorrelated variables, called factors, that explain the maximum amount of variance in the original variables. These factors can create an index or composite score, which can be useful for data reduction, visualization, and statistical analysis. In a previous study is conducted in Taiwan, PAF was used to create an index of the quality of life for older people. The authors found that PAF was effective in identifying the underlying dimensions of quality of life for the elderly, which was represented by multiple variables. PAF is used to determine main global indexes using several global databases. In total eight global social-related databases were used to determine the global social index.

Figure 1 presents all the important aspects of establishing an index using several global components by using statistical methods.

## 3. Results

### 3.1. Correlation between different social indicators and obesity

**Figure 2:**
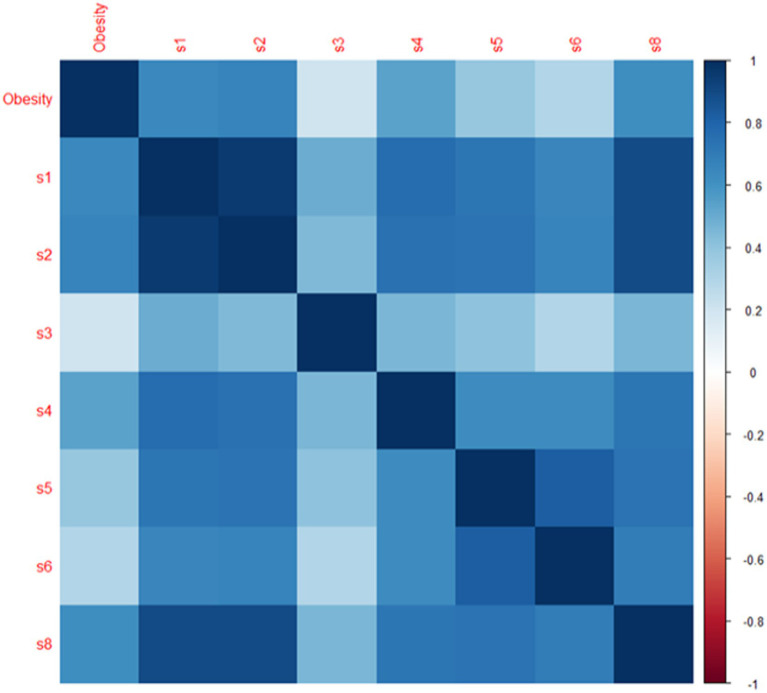
Correlation plot of global social indicators. *s1 represents human development index (%), s2 represents social globalization (%), s3 represents political globalization (%), s4 represents global happiness index (life ladder in %), s5 represents quality of overall infrastructures (1–7), s6 represents institutions (%), s7 represents individuals using the internet (% of population), s8 represents quality of roads (1–7).

Pearson correlation method has been used to check the relationship of all targeted social indicators and human obesity. Figure 2 shows the heat map depicting the correlation analysis between eight social indicators identified above and between obesity. The darkest blue color White and light blue, have neither shown any association between the desired global social indices nor exhibited higher correlations.

The correlation between worldwide society at a high human development level and global obesity has a substantial, positive, and direct relationship, indicating that global obesity is rising with an environment that is more educated in high human development countries.

Table 1 illustrates the internal correlations of international social indicators to examine precise global trends, significant effects, and variations. The results show that all the social indicators are positive and directly related with global human obesity. Almost all the global social indicators (human development index, social and political globalization, the global happiness index, and the standard of living overall infrastructures, institutions and individuals using the internet) have positive and direct relationships between them, but political globalization has observed a weak correlation.

**Table 1:** Correlation matrix of global human obesity and global social indicators.

	Global human obesity	Human development index	Social globalization	Political globalization	Global happiness index	Quality of overall infrastructures	Institutions	Individuals using the internet	Quality of roads
Global human obesity	1								
Human development index	0.6429	1							
Social globalization	0.6675	0.958	1						
Political globalization	0.2027	0.4911	0.4499	1					
Global happiness index	0.532	0.7626	0.7489	0.4506	1				
Quality of overall infrastructures	0.3823	0.7275	0.7352	0.4084	0.627	1			
Institutions	0.2956	0.6533	0.6638	0.2916	0.6218	0.8266	1		
Individuals using the internet	0.6191	0.8966	0.8949	0.4509	0.7231	0.7396	0.693	1	
Quality of roads	0.3279	0.6271	0.6374	0.4023	0.5706	0.9294	0.7731	0.6463	1

**Figure 3:**
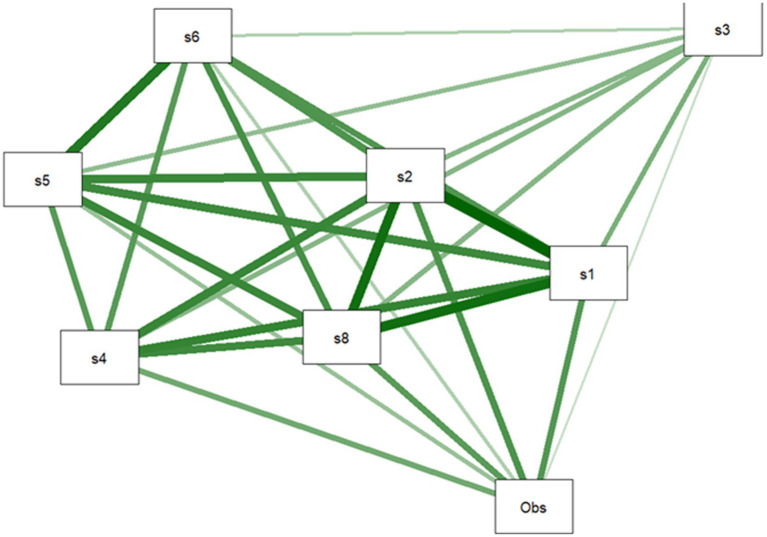
*Q*-graph of social indicators.

Figure 3 the Q-graph explains the connections between all social variables in their internal linkages. Strong correlations between the indications that are closest at hand and poor associations between the indicators that are farther away.

### 3.2. Determination of social index

The global social index is one of the essential factors in the universe that contain several variables that directly or indirectly affect human obesity. The following key statistical results of factor analysis are presented in Table 2.

**Table 2:** Results of social index.

Factors	Constructs	KMO	Chi-Sq	*p*-value	RMSR
Social index	8	0.88	917.7801	0.000	0.09

*KMO, Kaiser-Meyer-Olkin; Chi-sq, Chi-square test; RMSR, root mean square residual.

Table 2 presents two tests that determine whether your data is acceptable for structure detection. It is advised that you utilize the Kaiser-Meyer-Olkin sample adequacy measure to determine how much of the variance in your variables is attributable to underlying factors.

An example can be found in a study also used PAF to analyze the factor structure of a measure of student engagement. They found that a two-factor solution accounted for a large proportion of the total variance, and the factors were interpretable regarding behaviour and cognitive engagement. High values (around 1.0) imply that factor analysis would be advantageous for the given data set. The factor analysis results are probably not very informative if the value is <0.50. Considering the aforementioned findings, KMO, or root mean square of the residuals (RMSR) and *p*-values, indicate that the index is adequate and acceptable. Utilizing extraction communalities, one may estimate the variation in each variable that the elements of the factor solution take into account. Small value variables do not match the factor solution well and should be eliminated from the analysis. The variation described by the initial response is shown in Table 1.

The scree plot supports the choice to employ every component during line bends. The line becomes virtually flat after the second component, indicating that each subsequent component accounts for a smaller proportion of the overall variance. The study is only interested in maintaining primary components with eigenvalues larger than one.

**Figure 4:**
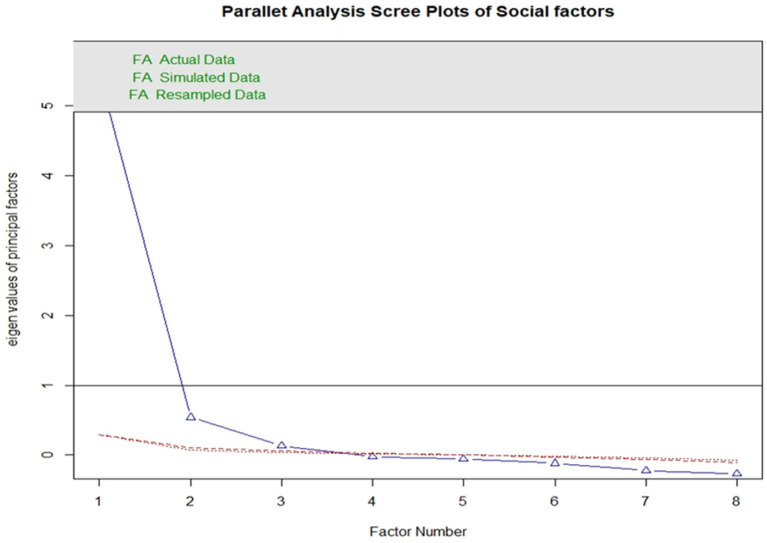
Scree plot for social indicators.

All social indicators are admissible to create a global social index with a cut point of 0.3 loading for each item. “Poverty headcount ratio at $1.90” has linear factorization, and principal component analysis of the component matrix revealed relatively low loading and more missing observations. In accordance with Table 1, the remaining datasets in all of them had appropriate KMO values. Using the extraction method through PAF, seven main global social indicators were used. The loading of 0.3 set criteria of a component was used so that two components were removed due to low loading. Finally, seven global indicators of society are used to create the index, which is created using exploratory factor analysis and principal axis factoring (Figure 4).

### 3.3. Relationship and clustering of global social index and global human obesity

The relationship between global human obesity and social index varies significantly relationship. An indirect relationship was observed in low and very high human development levels, but a positive correlation was seen between medium and high human development countries and global human obesity. With a value of 0.550, the correlation between the worldwide social index and the medium level of human development has a highly significant, positive, and direct association, demonstrating that global human obesity is rising as societies get more educated. With a value of 0.550, the correlation between the worldwide social index and the medium level of human development has a highly significant, positive, and direct association, demonstrating that global human obesity is rising as societies get more educated. At a very high level of human development, there is a considerable, adverse, weak, and indirect association between global human obesity and the global social index, with a −0.121 value. With a value of −0.078, the link between the global social index and the global prevalence of obesity is negligible, adverse, weak, and indirect.

**Figure 5:**
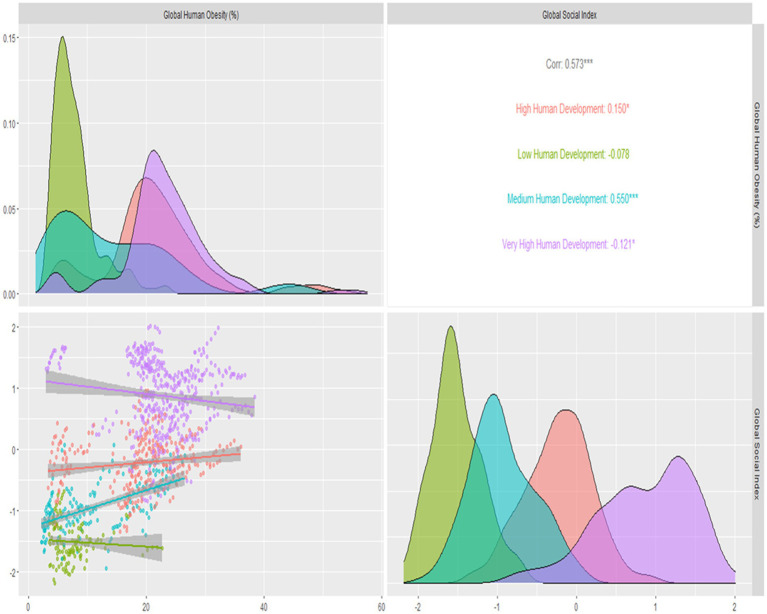
Correlations, density plots, clustering, trends, of global social and global human obesity by human development levels.

Figure 5 illustrates the relationship between worldwide social and human obesity about the degrees of global human development. It is a multi-objective and multi-dimensional visualization. The density plots with an interior fluctuation of data about the low, medium, high, and extremely high human development countries are explained in the following graphic. The following diagram also included scatter plots with trends for the four human development levels. Global human obesity and society are generally positively and directly correlated with one another. The findings show that by raising national social standards, worldwide human obesity is declining in levels of low, high, and very high human development (HDI), but the contrary pattern is observed in levels of medium and high HDI.

Table 3 presents the impact of all social indicators and the overall impact of the global social index on global human obesity. All the indicators of global social indicators are affecting human obesity except the human development index from a global perspective. Still, institutional quality plays a significant role in decreasing human obesity. The value of R-square is 0.53, representing that the social indicators are contributing 53% to development impact on global human obesity with 0.000 as a value of p with 1% significance. After making an index using all the above social indicators, the overall model is also significant. The global social index significantly contributes to increasing global human obesity. By Increasing one unit in the global social index, global human obesity is increasing 4.887 units on average. The overall model is also significant, with a value of *p* = 0.000 with a 1% level of significance.

**Table 3:** Regression model for global human obesity and global social indicators.

Variables	Estimate	Std. error	*t* value	Pr. (>|t|)	*R* square	*F*-statistics	*p* value
(Intercept)	8.16046	1.90823	4.276	0.0000 ***	0.53	139.3	0.0000 ***
Human development index	1.7155	4.78695	0.358	0.72014			
Social globalization	0.30325	0.04095	7.405	0.0000 ***			
Political globalization	−0.10652	0.01605	−6.638	0.0000 ***			
Global happiness index	1.34065	0.26875	4.989	0.0000 ***			
Quality of infrastructures	−1.38231	0.52287	−2.644	0.0083 **			
Institutions	−3.23441	0.3978	−8.131	0.0000 ***			
Individuals using the internet	0.08467	0.01595	5.31	0.0000 ***			
Quality of roads	1.038	0.41581	2.496	0.0127 *			
**Overall model**							
(Intercept)	18.1241	0.222	81.64	0.0000 ***	0.3289	487.6	0.0000 ***
Global social index	4.887	0.2213	22.08	0.0000 ***			

Significant. codes: 0 “***” 0.001 “**” 0.01 “*” 0.05 “.” 0.1.

**Figure 6:**
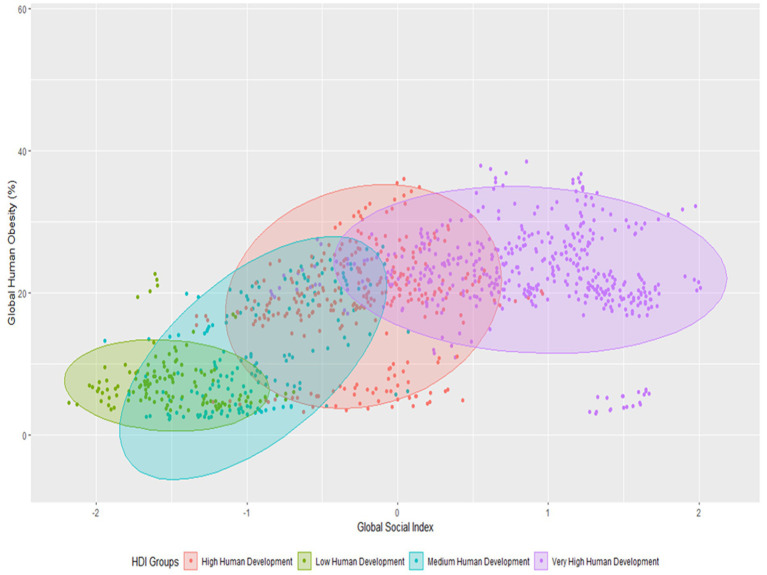
Clustering of global human obesity with global social index through HDI.

In Figure 6, global clusters of societal and human obesity have been created based on the human development levels of four different countries. Four quadratic techniques have been employed. Low social indicators and moderate obesity were present in low human development countries in the first phase, and moderate global obesity and low global social indicators were present in medium human development countries in the second phase. Third, countries with very high levels of human development also have high levels of social inequality and global obesity. In this image, it is demonstrated that countries with extremely high levels of human development have the highest levels of society and human obesity, while those with low levels of human development have the lowest levels of society and obesity.

## 4. Discussion

An increased energy level causes obesity in the body as a result of variables such as excessive energy consumption (through big portions or high-energy-density meals) or insufficient physical exercise. As seen by the high prevalence of obesity in Western industrial countries, such variables of the social index include the human development index, social globalization, global happiness index, quality of overall infrastructures, institutions and individuals using the internet are frequently a by-product of westernization and abundant economic wealth (40). Obesity prevention initiatives undertaken by the government are ineffective. The number of overweight people in the world is today much larger than the number of underweight people. Globalization is the integration of global economies, societies and cultures through a worldwide network of economic, technological, socio-cultural, political and biological forces (41).

Globalization has diverse consequences for different countries and regions. In comparison, most prior works focused on the link between globalization and obesity, and no specific quantitative measurements of the wide range of potentially very varied globalization-related mechanisms have been investigated previously (42). It is pertinent to study the effect of social globalization on global obesity spreading so enough data will be available for policymakers to develop effective controlling strategies. In this analysis, we have tried to find the relationship between obesity and different dimensions of social globalization.

After the initial shortlisting of seven different social variables, a global social index was constructed. Initially, each of these seven variables was correlated to check the exact global trends which depict positive and direct relationships between them. The relationship between global obesity was correlated with different levels of human development levels (28, 43). The results of this study showed that the obesity patterns significantly increase at the medium human development level. This can be analyzed by the fact that the medium development human index rises due to industrialization in developing countries, increased income, low physical activity, and variations in nutritional habits towards fast junk foods. This would appear to show that men and women are more likely to be overweight when the economy is doing well. The rationale here is that as a country’s economy shifts from being more agrarian to more industrialized, its food sector goes through a concomitant shift into more processed and fast foods linked to higher population obesity incidence (44).

Contrastingly an opposite relationship was observed between low human development levels and very high human development levels, with a negative correlation between them. This can be idealized by the fact the people living in rural areas (low human development level) do not have access to technology and still live the natural life of their generations. Traditional BMI was lower in rural communities, which had reduced food consumption due to poorer earnings and more energy expenditure in everyday work and home activities. Additionally, their less adaptability towards westernization results in far more physical activity than their counterparts who migrated to bigger cities for better economic prospects hence indicating a negative trend of obesity. We also observed a negative correlation between very high human development levels and obesity because people living in this category of the human index are more sensitive towards their health. This might be due to the increased awareness of a balanced diet and decreased consumption of high-caloric intakes in this sector. In addition, the routine practice of exercise in persons with high human development levels has been seen as a significant factor in reducing obesity levels.

## 5. Conclusion

Recently, behavioral scientists have emphasized that rather than continuing to develop purely individual-level therapies, policymakers should concentrate on the systemic causes of obesity. The study concludes a variation in correlation patterns between different kinds of human development levels and obesity which might help the policymakers of an individual country to monitor the progress of obesity amongst their nation to increase their progressiveness. This study shows an increase in obesity trends in the medium development human index countries. The results of our study showed that with the increase in Human development Index, Social Globalization and Individuals using the Internet, the trends in Human Obesity increases the most. This might be due to increased income of people living in these areas, where a transitional shift in economic developments due to rapid industrialization resulted in an increase in buying capacity and a more active, sedentary lifestyle. It is concluded that political globalization, quality of Infrastructure and institutions are playing a key role in reducing global obesity. In comparison, the human development index, social globalization, global happiness, internet usage and quality of roads are positively impacted to increase global obesity. The potential effect of society and culture on obesity should be addressed when designing interventions to reduce obesity for populations with specific cultural profiles.

## Data availability statement

The datasets presented in this study can be found in online repositories. The names of the repository/repositories and accession number(s) can be found in the article/supplementary material.

## Author contributions

MM and ZZ: conceptualization. MM, SK, and TE: methodology, project administration, and funding acquisition. HN and MM: software. MM, ZZ, HN, ZA, SK, and TE: validation, formal analysis, investigation, resources, data curation, writing – review and editing, visualization, and supervision. MM: writing – original draft preparation. All authors contributed to the article and approved the submitted version.

## Funding

The publication of this article was funded by the Open Access Fund of Leibniz Universität Hannover.

## Conflict of interest

The authors declare that the research was conducted in the absence of any commercial or financial relationships that could be construed as a potential conflict of interest.

## Publisher’s note

All claims expressed in this article are solely those of the authors and do not necessarily represent those of their affiliated organizations, or those of the publisher, the editors and the reviewers. Any product that may be evaluated in this article, or claim that may be made by its manufacturer, is not guaranteed or endorsed by the publisher.

## Footnotes

^1^ https://ourworldindata.org
